# Development of a rapid test to determine endometritis of cows after calving

**DOI:** 10.14202/vetworld.2024.2028-2035

**Published:** 2024-09-13

**Authors:** Isatay Jakupov, Axel Wehrend, Aida Abultdinova, Gulnur Mamytbekova, Zhanargul Zharkimbaeva, Albert Zabrodin

**Affiliations:** 1 Department of Veterinary Medicine, Faculty of Veterinary Medicine and Technology of Animal Husbandry, S. Seifullin Kazakh Agro Technical Research University, Astana, Kazakhstan; 2 Clinic for Obstetrics, Gynecology and Andrology of Large and Small Animals, Justus-Liebig University, Giessen, Germany

**Keywords:** cervicovaginal mucus, cows, laboratory diagnostics, peroxide test, endometritis

## Abstract

**Background and Aim:**

Postpartum uterine disease, such as endometritis, is widespread in highly productive dairy cows, leading to fertility problems and economic losses. Despite existing diagnostic methods, early and effective detection of uterine infections remains problematic due to the subclinical nature of some conditions. This study aimed to develop and evaluate a rapid diagnostic test for endometritis in cows on different days postpartum (PP).

**Materials and Methods:**

The study was carried out on dairy Holstein–Friesian cows (n = 121) kept on farms in northern Kazakhstan. The study included both clinically normal cows and those diagnosed with endometritis, ensuring a comprehensive evaluation of the diagnostic methods across different stages of uterine health. The following laboratory tests were performed to diagnose and evaluate the presence and severity of endometritis in the cows: Nagorny-Kalinovsky test, Whiteside test, Katerinov test, Gavrish silver nitrate test, peroxide test, and clinical tests (rectal and vaginal examination). These tests were specifically chosen to identify inflammatory processes in the uterus, particularly focusing on detecting endometritis.

**Results:**

From day 21 to 30 PP, rectal and vaginal examinations were 32% and 28% more effective than the Nagorny-Kalinovsky test and the Whiteside test. From day 61 postpartum, the Whiteside test was 37.5% more effective than the Nagorny-Kalinovsky test. Comparatively, among laboratory diagnostic methods from days 10 to 110 PP, the peroxide test showed the greatest effectiveness in identifying 80.9% of sick animals. In sick animals from day 10 to 20 PP, during the interaction of the cervicovaginal mucus with 10%, 20%, and 30% hydrogen peroxide (H_2_O_2_), an 8.1 ± 1.9–8.8 ± 1.6 cm foam column was formed within 4–5 min.

**Conclusion:**

The experiment showed that a 10% H_2_O_2_ solution yielded better results. Using H_2_O_2_ as a diagnostic agent for endometritis in cows has several advantages, including ease of use, it does not require special laboratory conditions and provides a visual reading of the reaction within 4–5 min. A limitation of this study is the focus on H_2_O_2_ without exploring other potential reagents that may enhance diagnostic accuracy. Future research could explore the long-term stability of cervicovaginal mucus samples and investigate the integration of additional substances that may expedite the detection of subclinical endometritis and improve the clarity of diagnostic results.

## Introduction

Despite the high reproductive rates in cows on large livestock farms due to genetics [1], fertility disorders remain a pressing concern [2], and uterine diseases in the uterus following calving are the primary cause [3–5]. Uterine diseases in the reproductive organs are most commonly observed in highly productive cows [6, 7]. According to Uzyntleuova *et al*. [8], endometritis is 28% more common in cows with high milk productivity than in less productive ones.

Uterine diseases of the reproductive system [9] most often turn into chronic forms (subclinical endometritis) [10], the prevalence of which ranges from 10.5% to 73% [7, 11]. Subclinical endometritis is one of the most difficult uterine diseases to diagnose [12], as it is asymptomatic and affects the reproductive and milk productivity of cows [13, 14]. In the case of untimely diagnosis and treatment of pathology of the reproductive system, these diseases result in significant economic losses due to a shift in the timing of insemination and lack of milk and offspring, followed by culling of animals [15]. The diagnosis of uterine diseases based on clinical [16], biophysical [17], and laboratory studies requires further study because these methods depend on the qualifications of the veterinarian and special skills required to conduct a qualitative study of an animal [18–20].

Endometritis diagnosis is carried out through the evaluation of cervicovaginal mucus (CVM) characteristics during the postpartum (PP) period [21], such as color, consistency, odor, and pH [22]. The complex biomaterial, CVM, protects the uterus with its various biological properties and could serve as a diagnostic tool for inflammatory conditions in the uterus [23–26]. The most common diagnostic method using CVM is the cytological method, which calculates polymorphonuclear neutrophils in the smear. However, laboratory conditions are necessary for cytological examination, which is extremely rare on dairy farms [27].

The urgent need for rapid diagnostic tests for chronic endometritis cannot be overstated. Our study concentrates on the utility of simple and effective rapid diagnostic tests for farmers. The rapid diagnostic test is based on the fact that the inflammatory products contained in CVM, for example, toxic substances of the aromatic series (indole and skatole), sulfur-containing amino acids, histamine, elevated protein, etc., when exposed to chemical reagents, show visible reactions in the form of turbidity or color changes, allowing one to determine uterine diseases [28]. The Dyudenko test [29] for diagnosing endometritis involves the examination of lochia or mucus collected during estrus from repeatedly inseminated cows. When the biological substrate interacts with a 20% solution of trichloroacetic acid, nitric acid, or 33% solution of caustic soda, with a positive reaction, the solution turns yellow, and the color saturation depends on the degree of inflammation [30]. According to the Popova test, the detection of leukocytes in cervical mucus is ensured by a change in the color of the liquid after adding an equal amount of 4% hydroxide solution. In the case of a negative reaction, the solution remains colorless. In the case of a positive reaction, the solution turns lemon yellow [31].

The novelty of this study lies in introducing hydrogen peroxide (H_2_O_2_) as a diagnostic agent for uterine diseases in cows, providing a rapid, cost-effective, and easily accessible test that yields visual results within 4–5 min. H_2_O_2_ was chosen as the agent for this study due to its well-known oxidative properties, which allow it to react with the enzymes in cervicovaginal mucus, leading to a visible foam formation that is easy to observe. The application of H_2_O_2_ specifically for the diagnosis of subclinical endometritis in cows is novel. This innovative approach addresses the challenge of detecting subclinical endometritis, a condition that significantly impacts fertility and milk production but is often asymptomatic and difficult to diagnose. By leveraging the chemical reaction between H_2_O_2_ and CVM to indicate inflammation through foam formation, the study offers a practical solution for on-farm diagnostics, in contrast to more complex and time-consuming traditional methods. Furthermore, the comprehensive evaluation of the peroxide test against established clinical and laboratory methods underscores its superior effectiveness in various postpartum periods, highlighting its potential to enhance dairy cow health management and productivity. By developing a rapid, easy-to-use diagnostic test utilizing H_2_O_2_, this research provides a cost-effective and accessible tool for early detection and treatment, enhancing both animal welfare and farm productivity.

This study aimed to determine the effectiveness of clinical and laboratory methods for diagnosing endometritis in cows on different days after calving and to evaluate the effectiveness of H_2_O_2_ at various concentrations as a rapid test for detecting endometritis.

## Materials and Methods

### Ethical approval

This study was approved by the ethics committee of S. Seifullin Kazakh Agrotechnical University in “Minutes No. 3 of the meeting of the local ethics commission on biological and medical ethics for research on an animal” dated November 3, 2022.

In the study preparation and planning, we adhered to high ethical standards related to animal welfare, such as the Consensus on Ethics and Animal Welfare of the International Association of Editors of Veterinary Journals (Geneva, Switzerland, 2010), and zoohygienic requirements (Law on Responsible Treatment of Animals of the Interparliamentary Assembly of the Commonwealth of Independent States [No. 46–15 dated March 27, 2017]) [32].

### Study period and location

The study was conducted from August 2023 to February 2024. Practical tests and sampling were performed at the agricultural formations of the Akmola and North Kazakhstan regions of the Republic of Kazakhstan (Rodina Agricultural Company LLP, Salyut LLP). Laboratory diagnostic methods were performed in the Laboratory of Obstetrics, Gynecology, and Reproduction Biotechnology at the Department of Veterinary Medicine, S. Seifullin Kazakh Agrotechnical University.

### Animal selection and examination

The prevalence range was derived from previous epidemiological studies on endometritis in dairy cows, as reported by Uzyntleuova *et al*. [8]. A sample size of 121 cows was chosen using the Cochran formula for sample size calculation in prevalence studies:


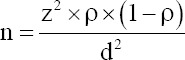


Where:

z is the Z-value (e.g., 1.96 for a 95% confidence level),

ρ is the estimated prevalence (using an average prevalence of 41.75% from the given range),

d is the desired precision (e.g., 0.1 for a 10% margin of error).

Out of 2,671 Holstein-Friesian cows, 121 cows of the 1^st^–5^th^ lactation aged 2–8 years were included in the study. The inclusion criteria for selecting cows in this study were as follows: Only Holstein–Friesian dairy cows aged between 2 and 8 years from the 1^st^ to the 5^th^ lactation were included. Cows with no significant health issues other than those related to the reproductive system were considered. The selected cows were at various stages postpartum, specifically targeting those within the first 110 days after calving and were from farms with loose and free-range management systems, ensuring consistent access to feed and water. Their diet consisted of a balanced mix of forages, concentrates, and supplements formulated to meet the nutritional requirements of lactating dairy cows [33]. This ensured that all animals received adequate nutrition to support their health and productivity throughout the study period [34, 35].

Each cow was registered with an identification number, and anamnesis was collected. The anamnesis noted the timing of the last calving, the course of labor, the presence of PP complications, and the results of a general external examination.

Based on an external examination, the fatness, body position, and symmetry of the pelvic bones were recorded, and the vulva and root of the tail were examined. In this study, the body condition scores of the cows ranged from 3 to 4, indicating that the cows were in good health and neither underweight nor overweight. In the presence of mucus secretions, their consistency and color were established; temperature, pulse, and respiration were determined.

Next, a clinical (gynecological) examination was performed, including transrectal and vaginal examinations, which served as a control diagnostic method. The transrectal examination determined the uterus topography, size, body, horns, cervix consistency, contractility, and fluctuations. During vaginal examination, CVM was collected manually (using gloves). The selected mucus was evaluated based on its color, odor, and consistency and placed in sterile containers for further laboratory examination.

The animals were divided into two groups based on different days of PP (Group-1: 67 heads and group-2: 54 heads). In group 1, the effectiveness of clinical (vaginal, rectal) and laboratory methods for the diagnosis of endometritis (Nagorny-Kalinovsky test, and the Whiteside test) was compared. Two experiments were carried out on group 2. The first series of experiments considered the chemical reactions of CVM collected from cows with endometritis (n = 54) with the addition of various concentrations of H_2_O_2_ (10, 20, and 30%), including the reaction time, i.e., foam formation (minutes) and the amount of foam (cm) (peroxide test). The second series included a comparison of clinical and laboratory tests (Katerinov test, Gavrish silver nitrate test, and peroxide test) for the diagnosis of cow endometritis (n = 47). In connection with therapeutic measures, seven of the 54 cows were excluded from the second series due to a lack of sufficient data during the examination. These were two cows with 41–50 days PP, two cows with 51–60 days PP, and three cows with 61 or more days of PP. The grouping of animals according to days of PP is shown in Table-1.

**Table-1:** Grouping of animals based on days of postpartum (PP).

Group 1 (n = 67): Comparative diagnosis			

PP (Number of animals)		Research methods	

		Clinical (rectal, vaginal)	
21–30 (n = 25)		Laboratory (Nagorny-Kalinovsky test and Whiteside test)	
31–60 (n = 25)			
61–90 (n = 8)			
91 or more days (n = 9)			

**Group 2 (n = 54)**			

**Experiment 1: Chemical reaction**		**Experiment 2: Comparative diagnostics**	
	
**PP (Number of animals)**	**Method**	**PP (Number of animals)**	**Research methods**

10–20 (n = 14)	Peroxide test	10–20 (n = 14)	Clinical - Rectal - Vaginal Laboratory - Katerinov test for uterine involution - Gavrish silver nitrate test - Peroxide test
21–30 (n = 8)		21–30 (n = 8)	
31–40 (n = 8)		31–40 (n = 8)	
41–50 (n = 8)		41–50 (n = 6)	
51–60 (n = 8)		51–60 (n = 6)	
61 or more days (n = 8)		61 or more days (n = 5)	

### Laboratory diagnostic methods

The experiment consisted in determining the effectiveness of laboratory diagnostic methods, such as Nagorny-Kalinovsky test, Whiteside test, Katerinov test for uterine involution, Gavrish silver nitrate test, and peroxide test, compared with clinical methods (rectal and vaginal methods), which also served as control methods. A laboratory method involving H_2_O_2_ (peroxide test) was proposed and evaluated in this study as a diagnostic agent for the first time.

### Diagnosis of subclinical endometritis according to Nagorny-Kalinovsky test

Two mL of vaginal mucus was poured into a test tube, and 2 mL of 1% acetic acid solution was added. The reaction was considered positive in the case of sediment formation and turbid sedimentary liquids [24].

### Whiteside test

1 mL of uterine secretions was mixed with 1 mL of a 5% sodium hydroxide solution, heated to a boil, and cooled with tap water. A positive reaction occurred when the resulting solution turned yellow [25].

### Katerinov test for uterine involution

3–5 mL of distilled water was poured into a test tube, and mucus from the cervix (a quantity the size of a pea) was added. The mixture was boiled for 1–2 min. With complete involution of the uterus, the fluid remained transparent, and with subinvolution, it became turbid with flakes [26].

### Garish silver nitrate test

The Gavrish silver nitrate test detects histamine in cow urine by mixing the urine with an aqueous solution of silver nitrate. A positive reaction is indicated by the formation of a black precipitate. The possibility of conducting such a study does not depend on the phase of the sexual cycle. 1 mL of 4% aqueous solution of silver nitrate was added to a test tube containing 2 mL of urine and gently boiled for 2 min on a gas burner. The appearance of a black precipitate indicates a positive reaction, whereas a brown or lighter precipitate indicates a negative reaction [27].

### Peroxide test

To obtain a quick test to determine inflammation in the cow uterus, the properties of CVM were studied, and the effectiveness of 10, 20, and 30% concentrations of H_2_O_2_ as diagnostic agents was analyzed. The peroxide test is based on determining enzyme activity in the mucus of sick animals. Description: 1 mL of CVM was poured into a glass tube, and 2 mL of H_2_O_2_ at various concentrations (10%, 20%, and 30%) was added. Within 4 min, foam formed in the mucus of cows with endometritis when the diagnostic agent was added.

### Statistical analysis

The obtained data were processed mathematically using Microsoft Excel 365 (Microsoft Office, Washington, USA). The effectiveness of clinical and laboratory diagnostic methods was determined by comparing the results. For the statistical analysis of group comparisons with covariate time, a one-way covariance analysis was performed using the BMDP statistical software package, BMDP1V software (BMDP Statistical Software; University of California, Los Angeles, USA).

## Results

The effectiveness of clinical and laboratory methods for diagnosing endometritis in cows on different days after calving was assessed, as detailed in Table-2.

**Table-2:** The effectiveness of clinical and laboratory methods for diagnosing endometritis in cows on different days after calving (n = 67).

Method	Postpartum days							

	21–30 (n = 25)		31–60 (n = 25)		61–90 (n = 8)		91 and more (n = 9)	
			
	n	%	n	%	n	%	n	%
Clinical								
Rectal	17	68	11	44	4	50	3	33.3
Vaginal	17	68	15	60	4	50	3	33.3
Laboratory								
Nagorny-Kalinovsky test	9	36	5	20	2	25	1	11.1
Whiteside test	10	40	12	48	5	62.5	6	66.7

**Figure-1:**
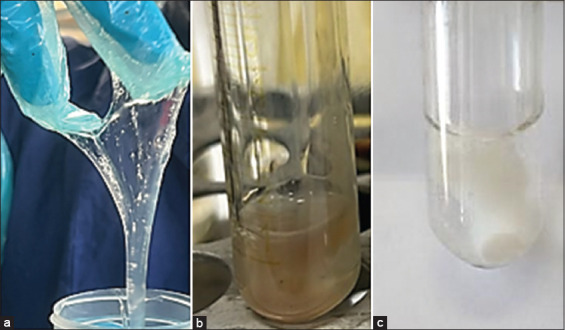
Nagorny-Kalinovsky test: (a) Selected cervicovaginal mucus. (b) positive reaction: sediment has formed, and the supravaginal fluid is turbid. (c) negative reaction: A clot was formed, and the supravaginal fluid was transparent.

**Figure-2:**
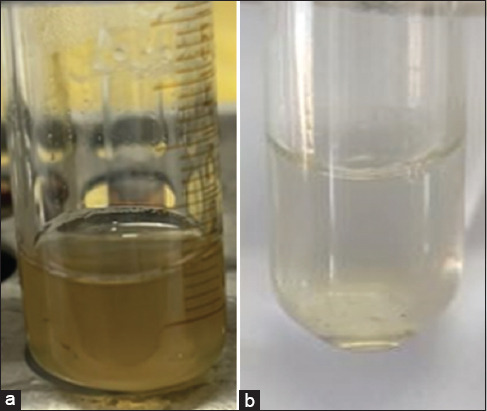
Whiteside test; positive reaction: (a) A lemon-yellow solution and (b)negative reaction: A colorless solution.

According to the results, 21–30 days after calving, rectal and vaginal examinations were 32% more effective than the Nagorny-Kalinovsky test (Figure-1) and 28% more effective than the Whiteside test (Figure-2). 31–60 days after calving, endometritis was effectively diagnosed with the vaginal examination, which was 1.3 times, or 16%, more sensitive than rectal examination, 3 times, or 40%, more sensitive than the Nagorny-Kalinovsky test and 1.25 times, or 12%, more sensitive than the Whiteside test. 61–90 days after calving, the vaginal examination was 11.8% more effective than the rectal examination. Laboratory studies using the Whiteside test showed 37.5% higher efficiency compared with the Nagorny-Kalinovsky test, and for clinical vaginal examination, the difference in efficiency was 12.5%. After 91 days, the Whiteside test was 33.4% more effective than vaginal and rectal examinations.

The results for group 2 are presented in Tables-3 and 4. Table-3 presents the chemical reaction of CVM in cows with endometritis with H_2_O_2_ at 10%, 20%, or 30% concentration. Table-4 presents data from a comparative analysis of the effectiveness of clinical and laboratory diagnostic methods.

**Table-3:** Chemical reaction of cervicovaginal mucus of cows with endometritis on the addition of different concentrations of H_2_O_2_ (n = 54).

Postpartum days	n	Reaction with H_2_O_2_					

		10%		20%		30%	
		
		reaction time, min	amount of foam, cm	reaction time, min	amount of foam, cm	reaction time, min	amount of foam, cm
10–20	14	4.5±1.5	8.6±1.5	4.5±1.5	8.8±1.6	5±1.9	8.1±1.9
21–30	8	4±0.5	6.3±1.9	4±0.7	6.2±1.3	4.2±0.5	5.8±1.2
31–40	8	4±0.7	2.25±0.7	4±0.5	2.4±0.9	4.2±0.9	1.3±0.3
41–50	8	4±1.2	1.7±0.3	4±0.7	1.15±0.6	4±1.2	1.5±0.3
51–60	8	4±1.3	5.1±0.7	4.6±1.2	5.3±1.95	4±1.3	5.7±2.34
61 and more	8	4.8±0.2	3.5±0.8	5±1.5	3.3±1.23	4.8±0.2	3.7±1.24

**Table-4:** Results of clinical and laboratory diagnostics of endometritis in cows on different days after calving (n = 47).

Postpartum days	n	Clinical methods				Laboratory methods					
	
		Rectal		Vaginal		Katerinov test		Gavrish silver nitrate test		10% H_2_O_2_ solution	
				
		n	%	n	%	n	%	n	%	n	%
10–20	14	11	78.6	12	85.7	3	21.4	2	14.3	14	100
21–30	8	7	87.5	8	100	5	62.5	3	37.5	7	87.5
31–40	8	2	25	5	62.5	4	50	1	12.5	6	75
41–50	6	1	16.7	3	50	3	50	4	66.7	4	66.7
51–60	6	1	16.7	3	50	1	16.7	1	16.7	2	33.3
61 and more	5	2	40	2	40	2	40	1	20	2	40
Total	47	24	51.1	33	70.2	18	38.3	12	25.5	38	80.9

**Figure-3:**
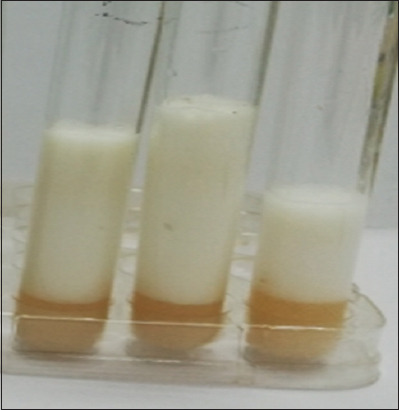
Reaction of cervicovaginal mucus with H_2_O_2_, foam formation.

According to Table-3, when CVM on 10–20 days PP interacted with H_2_O_2_ at 10, 20, and 30% concentrations, the foam was formed with a height of 8.6 ± 1.5, 8.8 ± 1.6, and 8.1 ± 1.9 cm, respectively. In the study of CVM on 21–30 days PP, the foam rose on average by 6.3 ± 1.9; 6.2 ± 1.3, and 5.8 ± 1.2 cm. From 31 days after calving, foam formation intensity was lower than in earlier periods. Foam formation was more intense on 10–20, 51–60, and 61 days after calving (Figure-3). Considering the average values, no significant deviations were observed when using 10%, 20%, and 30% H_2_O_2_ concentrations. For further experiments on the diagnosis of uterine inflammation, a 10% H_2_O_2_ concentration was adopted. To determine the effectiveness of the peroxide test, we used clinical methods (rectal and vaginal examination) and laboratory methods (Katerinov test, Gavrish silver nitrate test, and peroxide test). Table-4 presents the results of studies on cow mucus (n = 47) obtained during the clinical and laboratory diagnosis of uterine inflammation.

According to Table-4, during the examination of cows (n = 47) on days 10–110 after calving, the clinical rectal method identified 51.1% of cows with endometritis, and the vaginal method identified 70.2%. The percentage of uterine diseases determined using laboratory diagnostic methods was the following: 38.3% with the Katerinov test, 25.5% using the Gavrish silver nitrate test, and 80.9% using the peroxide test.

## Discussion

This is the first study to use H_2_O_2_ as a diagnostic agent for uterine inflammation in cows. We determined the effectiveness of clinical (rectal and vaginal) and laboratory (Nagorny-Kalinovsky test, Whiteside test, Katerinov test for uterine involution, and Gavrish silver nitrate test) diagnostic methods based on the chemical reaction of inflammatory products in the mucus and urine of sick animals interacting with chemical reagents.

In group 1, when examining the uterus from day 21 to day 60 of PP, we found that the rectal and vaginal methods were more effective. From 61 to 90 days of PP, the Whiteside laboratory method was 37.5% more effective than the Nagorny-Kalinovsky test, and from day 91, it was 33.4% more effective than the vaginal and rectal methods. Thus, clinical diagnostic methods were effective for acute endometritis within the first 2 months after calving. Laboratory methods allowed us to determine from 11.1% to 66.7% of the pathology cases that require further investigation.

In group 2, we observed that when 10%, 20%, or 30% H_2_O_2_ solution interacted with CVM, foam formed with an average height of 8.6 ± 1.5 cm. Foam formation in interaction with the mucus of sick animals was more intense from 10 to 20, 51 to 60, and 61 or more days after calving. The results showed that the optimal concentration of H_2_O_2_ solution for diagnosing uterine inflammation was 10%.

Several studies [36, 37] have been conducted on the chemical reactions of H_2_O_2_ with CVM and the enzymatic activity of mucus in healthy and sick animals.

Foam formation is based on the interaction of H_2_O_2_ with the enzyme catalase in body fluids. Catalase is an oxidoreductase enzyme that is part of the antioxidant system of cells and performs antiperoxidation protection [37]. Catalase activity is a significant indicator of the activity of the antioxidant system in terms of antiperoxide protection [38, 39]. Thus, in this study, H_2_O_2_ was used as a diagnostic agent, and the inflammatory process in the uterus was shown in terms of the intensity of foam column formation when H_2_O_2_ was added to CVM. In this study, by visually evaluating the reaction, we did not observe any change in the color of the solution or precipitation [40]. The standard liquid consists of sodium dodecyl sulfate, distilled water, and bromocresol purple, and its pH is regulated by sodium hydroxide. The coloring liquid consists of eosin, methanol, glacial acetic acid, distilled water, and sodium tetraborate mixed in a volume ratio. Despite the authors’ statements about the convenience and low cost of the detecting solution, we should note its complex composition [41, 42]. Using H_2_O_2_ as a diagnostic agent for endometritis in cows PP, considering that in this case, one does not need to prepare a complex diagnostic solution, remains a convenient and cost-effective laboratory method for farms.

## Conclusion

We recommend using the peroxide test at a 10% H_2_O_2_ concentration from 10 days onwards of PP. Using H_2_O_2_ as a diagnostic agent for endometritis in cows has several advantages, including ease of use. It does not require special laboratory conditions and renders a visual reading of the reaction within 4–5 min. Since there are no early data on conducting similar experiments, there are some issues that require further research, for example, how long mucus can be used when considering the reaction with H_2_O_2_, the possibility of including substances in the peroxide test that affect the accelerated detection of subclinical endometritis, and color changes with a positive reaction.

## Authors’ Contributions

IJ: Conceptualization, methodology, and writing - original draft preparation. AW: Supervision, validation, and writing - review and editing. AA: Data curation, investigation, and writing - review and editing. GM: Formal analysis, visualization, and writing - original draft. ZZ: Resources, project administration, and writing - review and editing. AZ: Software, data curation, and writing - review and editing. All authors have read, reviewed, and approved the final manuscript.
